# Associations between different triglyceride glucose index-related obesity indices and sarcopenia: a cross-sectional study

**DOI:** 10.3389/fendo.2025.1511232

**Published:** 2025-05-29

**Authors:** Wentao Xiao, Taichuan Xu, Yitao Liao, Yenan Xu, Zhihong Fan, Chao Li, Xian Zhang

**Affiliations:** ^1^ Department of Graduate School, Nanjing University of Chinese Medicine, Nanjing, China; ^2^ Department of Spine, Wuxi Affiliated Hospital of Nanjing University of Chinese Medicine, Wuxi, China

**Keywords:** NHANES, cross-sectional study, sarcopenia, TyG index, TyG-related obesity index

## Abstract

**Background:**

The relationship of triglyceride glucose (TyG) index-related obesity indices with sarcopenia has not been studied in the U.S. population.

**Methods:**

This cross-sectional survey utilizes data collected from the National Health and Nutrition Examination Survey spanning 2011 to 2018. The correlation between TyG-waist-to-height ratio (TyG-WHtR), TyG-weight-adjusted waist index (TyG-WWI), TyG-waist circumference (TyG-WC), TyG-body mass index (TyG-BMI), and sarcopenia was analyzed by weighted multivariate logistic regression using smoothed curves fitted to the observed nonlinear relationships and subgroup analysis were performed as well as an interaction test. Ultimately, the diagnostic validity of the four indices was evaluated in comparison with the TyG index for sarcopenia utilizing receiver operating characteristic (ROC) analysis.

**Results:**

This study included a total of 4804 participants, with 428 of those diagnosed with sarcopenia. The study illustrated that TyG-WHtR, TyG-WWI, TyG-BMI, and TyG-WC demonstrated a notable positive association with odds of sarcopenia prevalence [TyG-WHtR: OR (95% CI): 1.51(1.33, 1.72); TyG-WWI: OR (95% CI): 1.21 (1.18, 1.24); TyG-BMI: OR (95% CI): 1.15 (1.13, 1.18), TyG-WC: OR(95%CI):1.70(1.55, 1.87)]. Compared to the lowest quartile, the top quartile had a higher prevalence of sarcopenia, such as TyG-WWI (OR= 81.89, 95% CI: 38.49, 174.22). Subgroup analysis uncovered notable disparities in the relationship between four indices with sarcopenia., across gender and BMI≥30 strata. The association between the four distinct indices and sarcopenia is nonlinear. The ROC analysis demonstrated that four indices exhibited superior diagnostic efficacy to TyG. In addition, the diagnostic validity of TyG-WWI was optimal [TyG: AUC(95%CI):0.668(0.642,0.694); TyG-WHtR: AUC(95%CI):0.714(0.688,0.739); TyG-WC: AUC(95%CI):0.715(0.691,0.739); TyG-BMI: AUC(95%CI):0.737(0.714,0.761); TyG-WWI: AUC (95%CI):0.802(0.783,0.821)].

**Conclusions:**

TyG-WWI, TyG-WHtR, TyG-BMI, and TyG-WC are significantly positively linked to the prevalence of sarcopenia and outperform the TyG index in predicting this disorder. The TyG-related obesity indices, specifically the TyG-WWI index, have potential as reliable indicators for assessing and predicting sarcopenia.

## 1 Introduction

Sarcopenia, clinically characterized by progressive deterioration of skeletal muscle mass and functional capacity (1), has emerged as a critical global health challenge with substantial socioeconomic implications (2). From the age of 35, the human body experiences a gradual decline in muscle mass, with an approximate rate of 1-2% per year. After the age of 65, this rate of loss accelerates significantly, reaching up to 3% per year (3). This suggests that the risk of declining muscle quality and function commences in early adulthood, thereby further exacerbating the risk of muscle dysfunction in later life. While geriatric sarcopenia has received considerable scientific attention, muscle mass loss in younger populations remains underrecognized in clinical practice. Modern lifestyles (such as sedentary lifestyles and high-fat diets) have precipitated an epidemic surge in adiposity among younger cohorts (4, 5), and the coexistence of obesity and low muscle mass may synergistically exacerbate metabolic homeostasis disruption (6). Mounting evidence underscores the interplay between sarcopenic progression and metabolic dysregulation (7). Ongoing intensive research into sarcopenia has revealed insulin resistance (IR) as a significant factor in its progression, and simultaneously obesity can exacerbate IR and muscle atrophy (8). Consequently, the investigation of more accurate obesity-related markers of IR is essential for the timely identification of IR and the prevention of sarcopenia.

The TyG index is widely acknowledged as an efficacious and cost-effective marker for assessing IR, calculated from triglycerides and fasting glucose levels (9, 10). Recent studies have indicated that obesity indices associated with the triglyceride glucose(TyG) index offer a more precise evaluation of IR, such as the TyG-waist-to-height ratio (TyG-WHtR) (11, 12), as well as accurately reflect the risk of periodontitis, cardiovascular, diabetes, and other diseases (13–15). Prior research has established a connection between the TyG index and reduced muscle mass among the Korean population, as well as a positive correlation between the TyG index and sarcopenia among the US population (16, 17). Nevertheless, there is a paucity of studies investigating the correlation between different TyG index-related obesity indicators and sarcopenia.

Accordingly, this present study represents the first investigation into the correlation between multiple TyG index-related obesity indicators and sarcopenia, utilizing data obtained from The National Health and Nutrition Examination Survey (NHANES). Furthermore, the comparative analysis of TyG index-related obesity indicators with conventional TyG indices is undertaken with a view to identifying the optimal diagnostic efficacy, and thus providing a novel rationale to inform the prevention and evaluation of sarcopenia.

## 2 Methods

### 2.1 Study population

**Figure 1:**
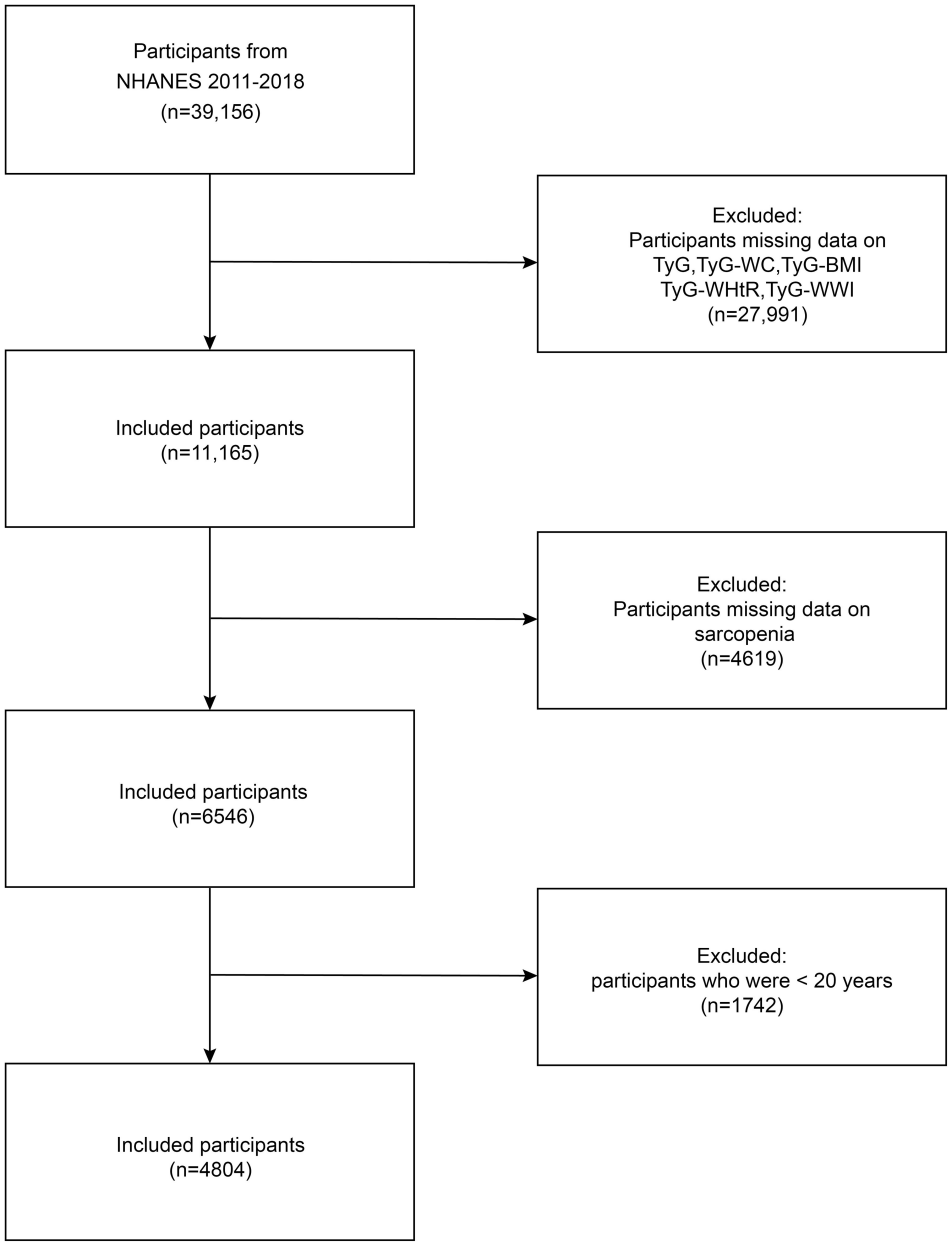
Flow chart of participants selection. NHANES, National Health and Nutrition Examination Survey, TyG-BMI, triglyceride glucose-body mass index, TyG-WC, triglyceride glucose-waist circumference, TyG-WHtR, triglyceride glucose-waist to height ratio, TyG-WWI, triglyceride glucose-weight-adjusted-waist index.

NHANES provides data on a range of nutritional intake, health status, and lifestyle factors within the United States, encompassing both adults and children. The sampling methods employed are sophisticated and are designed to ensure the sample population is effectively representative of the overall population (18). Before data collection, NHANES obtained approval from the NCHS Ethics Review Board and informed consent from all participants. This research gathered data from 39,156 participants across four cycles of the NHANES conducted between 2011 and 2018. The following participants were excluded from the study: those who lacked fasting blood glucose, triglyceride, height, weight, and waist circumference data; those who could not determine the muscle mass data of the limbs by dual-energy X-ray absorptiometry (DXA); and those younger than 20 years of age. Ultimately, the study included 4,804 individuals. The process of data selection and exclusion is illustrated in **Figure 1**.

### 2.2 Definition and calculation of TyG and related obesity indicators

The TyG index is calculated from fasting blood glucose and triglyceride levels to quantify insulin resistance. Participants were required to fast for a period exceeding 8.5 hours, and blood samples were collected in the early hours of the morning and dispatched to the central laboratory where fasting glucose and triglyceride levels were measured. A comprehensive description of the laboratory procedures can be found on the official NHANES website (https://wwwn.cdc.gov/nchs/nhanes/). In addition, waist circumference, height, and weight were measured at the mobile examination center. The formula below is utilized to compute the TyG index: ln[triglyceride levels(mg/dL)×fasting glucose levels(mg/dL)/2]. Based on previous studies (14, 15), use this index to calculate other relevant obesity indices. WC was the waist circumference (cm); Body mass index(BMI)=weight(kg)/height^2^(m^2^); WHtR=WC(cm)/Height(cm); WWI=WC(cm)/√weight(kg); TyG-WHtR=TyG×WHtR;TyG-WWI=TyG×WWI; TyG-WC=TyG×WC; TyG-BMI=TyG×BMI.

### 2.3 Assessment of sarcopenia

The appendicular skeletal muscle mass (ASM) represents the skeletal muscle mass of the extremities, as determined by dual-energy X-ray absorptiometry (DXA). The DXA test was performed using the Hologic QDR-4500A fan-ray densitometer, and subjects were not required to fast before the test but were required to remove metal objects from their personal belongings and wear clothing without metal accessories. Pregnant women, those with a body weight greater than 136 kg, or those with a length greater than the range measured by the device were excluded. The definition of sarcopenia is derived from the guidelines established by the Foundation for the National Institutes of Health (FNIH). The sarcopenia index, calculated as ASM/BMI, is utilized to assess sarcopenia. A sarcopenia index of <0.512 for women and <0.789 for men is indicative of sarcopenia (19).

### 2.4 Covariates

The following covariates were included: gender, age, race, education level, poverty income ratio (PIR) smoking status, drinking status, hypertension, diabetes mellitus, moderate physical activity, triglycerides, and fasting glucose. **Supplementary Table 1** provides definitions of smoking status, drink status, hypertension, diabetes, and moderate physical activity.

### 2.5 Statistical analysis

The statistical analysis used the R software (version 4.2.0) and the Epowerstats software. Statistical significance was determined using a *P*-value of <0.05. This research considered the intricate sampling design of NHANE and employed the recommended methodology for calculating sample weights to adjust the data. The weights utilized were as follows: WTMEC2YR/4. The relationships between four distinct TyG-related indices of obesity with sarcopenia were investigated by weighted multivariate logistic regression modeling, in which TyG-related obesity indices were both continuous and categorical variables. The present study employed three distinct adjustment models for its analytical procedures. Model 1 did not incorporate the use of covariates while Model 2 incorporates adjustments for gender, race, and age. Model 3 incorporates adjustments across all covariates. The non-linear relationship between the TyG-related obesity index and sarcopenia was investigated through the use of smoothed curve fitting. The subgroup analysis was also performed to investigate the effects of gender, age, BMI, diabetes mellitus, hypertension, and moderate to moderate physical activity, with further interaction tests. Furthermore, the diagnostic effectiveness of TyG, four distinct TyG-related indices of obesity in identifying sarcopenia through receiver operating characteristic (ROC) curve analysis, determining the area under the curve (AUC) to assess the sensitivity and specificity in predicting sarcopenia. Then, the area under the ROC curve of four different TyG-related obesity indices and the TyG index were compared by using the method of DeLong et al. to observe whether these indices had statistically significant differences and which index had the best predictive ability (20). Finally, The Benjamini-Hochberg (BH) method was employed to perform false discovery rate (FDR) correction on the *P*-value obtained by using the method of DeLong et al. and to observe whether the corrected result was significant.

## 3 Results

### 3.1 Baseline characteristics

**Table 1** presents the fundamental characteristics of the participants. The study included 4,804 participants, among whom 428 met the criteria for sarcopenia, representing a prevalence of 8.91%. The mean age(95% CI) of the sarcopenic individuals was 39.57(39.03, 40.10), and the mean BMI was 28.64(28.32, 28.96). In comparison to non-sarcopenic patients, those with sarcopenia are distinguished by a higher age, a higher BMI, elevated fasting glucose and triglyceride levels, and a lower PIR and educational attainment. The prevalence of sarcopenia is elevated in men, Mexican Americans, individuals who consume alcohol, smokers, those with elevated blood pressure, diabetics, and those who do not engage in moderate physical activity.

**Table 1:** The basic characteristics of the participants.

Characteristics	Total Mean(95%CI)	Non-sarcopenia Mean(95%CI)	Sarcopenia Mean(95%CI)	*P*-value
n	4804	4376	428	
Age(years)	39.57 (39.03,40.10)	39.24 (38.67,39.81)	43.62 (42.12,45.12)	<0.0001
Gender (%)				0.2090
Male	50.65 (48.73,52.57)	50.32 (48.23,52.40)	54.82 (48.27,61.21)	
Female	49.35 (47.43,51.27)	49.68 (47.60,51.77)	45.18 (38.79,51.73)	
Race (%)				<0.0001
Mexican American	10.59 (8.47,13.15)	9.45 (7.52,11.80)	24.85 (19.87,30.60)	
Other Hispanic	7.51 (5.99,9.37)	7.05 (5.58,8.88)	13.23 (9.43,18.25)	
Non-Hispanic White	61.74 (57.82,65.51)	62.71 (58.76,66.50)	49.57 (42.73,56.42)	
Non-Hispanic Black	10.60 (8.86,12.65)	11.22 (9.37,13.39)	2.85 (1.74,4.65)	
Other Race	9.56 (8.26,11.05)	9.57 (8.23,11.09)	9.50 (6.47,13.73)	
Body mass index (%)	28.64 (28.32,28.96)	28.17 (27.87,28.48)	34.54 (33.65,35.42)	<0.0001
Poverty income ratio (%)	2.83 (2.72,2.95)	2.88 (2.76,3.00)	2.28 (2.08,2.48)	<0.0001
Triglyceride (mg/dl)	120.22 (115.37,125.06)	117.40 (112.66,122.14)	155.48 (141.73,169.23)	<0.0001
Glucose (mg/dl)	104.45 (103.26,105.65)	103.40 (102.27,104.54)	117.59 (112.77,122.41)	<0.0001
Education level (%)				<0.0001
Less than high school	13.96 (12.06,16.11)	12.83 (11.02,14.89)	28.09 (22.34,34.68)	
High school diploma	21.58 (19.39,23.94)	21.38 (19.09,23.87)	24.03 (19.57,29.14)	
More than high school	64.44 (61.02,67.73)	65.77 (62.21,69.16)	47.87 (42.10,53.70)	
Others	0.02 (0.00,0.12)	0.02 (0.00,0.13)	0.00 (0.00,0.00)	
Hypertension status (%)				<0.0001
Yes	23.40 (21.50,25.42)	22.46 (20.60,24.43)	35.24 (29.16,41.85)	
No	76.60 (74.58,78.50)	77.54 (75.57,79.40)	64.76 (58.15,70.84)	
Smoking status (%)				0.8338
Yes	42.21 (39.65,44.82)	42.16 (39.49,44.88)	42.86 (36.65,49.31)	
No	57.79 (55.18,60.35)	57.84 (55.12,60.51)	57.14 (50.69,63.35)	
Drinking status (%)				<0.0001
Yes	79.83 (77.80,81.72)	80.52 (78.49,82.40)	71.19 (65.74,76.09)	
No	20.17 (18.28,22.20)	19.48 (17.60,21.51)	28.81 (23.91,34.26)	
Diabetes status (%)				<0.0001
Yes	5.84 (4.99,6.83)	5.20 (4.41,6.12)	13.92 (9.94,19.15)	
No	94.16 (93.17,95.01)	94.80 (93.88,95.59)	86.08 (80.85,90.06)	
Moderate physical activity (%)				<0.0001
Yes	47.77 (45.49,50.06)	49.03 (46.69,51.37)	32.08 (26.88,37.76)	
No	52.23 (49.94,54.51)	50.97 (48.63,53.31)	67.92 (62.24,73.12)	
TyG-BMI quartile				<0.0001
Q1	24.86 (22.79,27.06)	26.50 (24.32,28.80)	4.36 (3.13,6.06)	
Q2	25.69 (24.13,27.31)	26.75 (25.13,28.44)	12.39 (8.67,17.40)	
Q3	24.94 (23.10,26.88)	25.03 (23.19,26.96)	23.86 (17.75,31.26)	
Q4	24.50 (22.61,26.50)	21.72 (19.91,23.65)	59.39 (51.63,66.71)	
TyG-WC quartile				<0.0001
Q1	24.16 (22.24,26.18)	25.79 (23.77,27.91)	3.74 (2.39,5.80)	
Q2	25.21 (23.84,26.64)	25.99 (24.56,27.48)	15.43 (11.32,20.68)	
Q3	25.36 (23.79,26.98)	25.29 (23.77,26.87)	26.18 (19.74,33.83)	
Q4	25.28 (23.30,27.36)	22.93 (21.13,24.84)	54.65 (46.88,62.20)	
TyG-WHtR quartile				<0.0001
Q1	26.68 (24.63,28.84)	28.24 (26.07,30.51)	7.21 (4.81,10.67)	
Q2	26.50 (24.90,28.17)	26.77 (25.19,28.41)	23.13 (18.26,28.83)	
Q3	25.18 (23.26,27.20)	25.16 (23.20,27.23)	25.36 (20.29,31.20)	
Q4	21.64 (19.84,23.56)	19.83 (18.00,21.79)	44.30 (37.77,51.04)	
TyG-WWI quartile				<0.0001
Q1	25.36 (23.27,27.56)	27.28 (25.11,29.56)	1.28 (0.72,2.28)	
Q2	25.49 (23.91,27.15)	26.93 (25.30,28.63)	7.48 (4.95,11.15)	
Q3	25.20 (23.41,27.08)	25.24 (23.38,27.20)	24.66 (19.39,30.82)	
Q4	23.95 (21.89,26.14)	20.55 (18.66,22.57)	66.57 (60.38,72.24)	

For continuous variables: survey-weighted mean (95% CI), *P*-value was by survey-weighted linear regression. For categorical variables: survey-weighted percentage (95% CI), *P*-value was by survey-weighted Chi-square test. TyG-BMI, triglyceride glucose-body mass index; TyG-WC, triglyceride glucose-waist circumference; TyG-WHtR, triglyceride glucose-waist to height ratio; TyG-WWI, triglyceride glucose-weight-adjusted-waist index.

In order to obtain a comprehensive overview of the discrepancies in the distribution of each parameter, the individual indices were classified into quartiles. As demonstrated in **Supplementary Tables 2**–**5**, the quartiles with the highest TyG-related obesity indices demonstrated a higher prevalence of sarcopenia than the lowest.

### 3.2 Association between TyG-related obesity indicators and sarcopenia

As presented in **Table 2**, Model 3 revealed a significant positive correlation between the TyG-WWI, TyG-WHtR, TyG-BMI, and TyG-WC and the prevalence of sarcopenia after controlling for potential confounding factors[TyG-WHtR: OR (95% CI): 1.51 (1.33, 1.72); TyG-WWI: OR (95% CI): 1.21 (1.18, 1.24); TyG-BMI: OR (95% CI): 1.15 (1.13, 1.18); TyG-WC: OR (95% CI): 1.70 (1.55, 1.87)]. The prevalence of sarcopenia was 13.96 and 81.89 times higher in the highest quartile of TyG-BMI and TyG-WWI, respectively, compared to the lowest quartile (TyG-BMI: OR=13.86,95% CI:9.05, 21.23; TyG-WWI: OR=81.89,95% CI:38.49, 174.22). Furthermore, the examination of the trend showed that all indices were statistically significant (all *P* for trend < 0.0001).

**Table 2:** Associations between TyG-related obesity indices and sarcopenia.

Sarcopenia	(n) % (95%CI)	Model 1	Model 2	Model 3
		OR (95%CI) *P*-value	OR (95%CI) *P*-value	OR (95%CI) *P*-value
TyG-BMI	(4804) 7.39(6.13,8.65)	1.14 (1.12, 1.15) <0.0001	1.14 (1.12, 1.16) <0.0001	1.15 (1.13, 1.18) <0.0001
TyG-BMI quartile				
Q1	(1201) 1.30(0.83,1.77)	Ref.	Ref.	Ref.
Q2	(1201) 3.56(2.37,4.75)	2.81 (1.70, 4.66) 0.0002	2.29 (1.35, 3.89) 0.0034	2.26 (1.33, 3.85) 0.0044
Q3	(1201) 7.07(4.61,9.53)	5.79 (3.78, 8.87) <0.0001	4.36 (2.77, 6.84) <0.0001	4.17 (2.64, 6.60) <0.0001
Q4	(1201) 17.92(14.75,21.09)	16.61 (11.26, 24.48) <0.0001	14.08 (9.72, 20.41) <0.0001	13.86 (9.05, 21.23) <0.0001
*P* for trend		<0.0001	0.0016	<0.0001
TyG-WC	(4804) 7.39(6.13,8.65)	1.58 (1.48, 1.68) <0.0001	1.57 (1.48, 1.67) <0.0001	1.70 (1.55, 1.87) <0.0001
TyG-WC quartile				
Q1	(1201) 1.14(0.60,1.69)	Ref.	Ref.	Ref.
Q2	(1201) 4.52(3.22,5.83)	4.10 (2.40, 6.99) <0.0001	3.42 (1.99, 5.86) <0.0001	3.29 (1.90, 5.69) 0.0001
Q3	(1201) 7.63(5.04,10.22)	7.14 (4.01, 12.71) <0.0001	5.67 (3.24, 9.91) <0.0001	5.30 (3.03, 9.26) <0.0001
Q4	(1201) 15.98(13.19,18.77)	16.44 (10.04, 26.91) <0.0001	13.76 (8.82, 21.46) <0.0001	12.36 (7.69, 19.88) <0.0001
*P* for trend		<0.0001	<0.0001	<0.0001
TyG-WHtR	(4804) 7.39(6.13,8.65)	1.61 (1.47, 1.75) <0.0001	1.57 (1.40, 1.76) <0.0001	1.51 (1.33, 1.72) <0.0001
TyG-WHtR quartile				
Q1	(1201) 2.00(1.13,2.86)	Ref.	Ref.	Ref.
Q2	(1201) 6.45(4.51,8.39)	3.38 (2.07, 5.54) <0.0001	3.06 (1.86, 5.03) <0.0001	3.05 (1.85, 5.03) 0.0001
Q3	(1201) 7.45(5.76,9.13)	3.95 (2.42, 6.43) <0.0001	3.18 (1.97, 5.14) <0.0001	3.01 (1.88, 4.82) <0.0001
Q4	(1201) 15.13(12.00,18.27)	8.75 (5.43, 14.10) <0.0001	7.21 (4.28, 12.15) <0.0001	6.28 (3.61, 10.91) <0.0001
*P* for trend		<0.0001	<0.0001	<0.0001
TyG-WWI	(4804) 7.39(6.13,8.65)	1.10 (1.09, 1.11) <0.0001	1.09 (1.08, 1.10) <0.0001	1.21 (1.18, 1.24) <0.0001
TyG-WWI quartile				
Q1	(1201) 0.37(0.15,0.60)	Ref.	Ref.	Ref.
Q2	(1201) 2.17(1.33,3.01)	5.91 (2.94, 11.88) <0.0001	5.01 (2.45, 10.27) 0.0001	5.34 (2.56, 11.16) 0.0001
Q3	(1201) 7.23(5.22,9.25)	20.80 (11.65, 37.15) <0.0001	16.87 (9.37, 30.35) <0.0001	20.27 (10.69, 38.42) <0.0001
Q4	(1201) 20.55(17.09,24.00)	68.98 (36.56, 130.15) <0.0001	53.68 (28.07, 102.68) <0.0001	81.89 (38.49, 174.22) <0.0001
*P* for trend		<0.0001	<0.0001	<0.0001

95%CI: survey-weighted percentage, For sarcopenia: survey-weighted OR (95%CI) *P*-value. Model 1 did not incorporate the use of covariates. Model 2 incorporates adjustments for gender, age, and race. Model 3 incorporates adjustments for race, education level, triglyceride, glucose, poverty income ratio, hypertension, smoking, drinking, and moderate physical activity. TyG–BMI was treated as a continuous variable with per 10-unit increase. TyG–WC was treated as a continuous variable with a per 100-unit increase. TyG-BMI, triglyceride glucose-body mass index; TyG-WC, triglyceride glucose-waist circumference; TyG-WHtR, triglyceride glucose-waist to height ratio; TyG-WWI, triglyceride glucose-weight-adjusted-waist index, Ref., reference.

### 3.3 Subgroup analysis

Subgroup analysis and interaction tests were performed for gender, age, BMI, diabetes and hypertension, and moderate physical activity, as illustrated in **Table 3**. The correlations between TyG-BMI, TyG-WHtR, and TyG-WWI and sarcopenia differed in stratification by gender, age, BMI ≥30, diabetes mellitus, hypertension, and moderate physical activity (*P*<0.05). The correlations of TyG-BMI, TyG-WHtR, and TyG-WWI with sarcopenia were more significant in the stratification of gender and BMI ≥30 (*P* for interaction<0.05).

**Table 3:** Subgroup analysis for the association between TyG-related obesity indices and sarcopenia.

Subgroups	TyG-BMI	*P* for interaction	TyG-WC	*P* for interaction	TyG-WHtR	*P* for interaction	TyG-WWI	*P* for interaction
	OR (95%CI) *P*-value		OR (95%CI) *P*-value		OR (95%CI) *P*-value		OR (95%CI) *P*-value	
Gender		<0.0001		<0.0001		0.0119		<0.0001
Male	1.19 (1.07, 1.32) 0.0024		1.14 (0.98, 1.34) 0.0981		8.67 (6.40, 11.74) <0.0001		1.24 (1.19,1.29) <0.0001	
Female	1.09 (0.99, 1.21) 0.0764		0.81 (0.68, 0.97) 0.0282		6.73 (5.19, 8.71) <0.0001		1.14 (1.11,1.17) <0.0001	
Age		0.5035		0.6714		0.5580		0.2891
<40	1.08 (1.01, 1.14) 0.0197		1.05 (0.89, 1.24) 0.5843		3.49 (2.98, 4.08) <0.0001		1.08 (1.06,1.10) <0.0001	
>40	1.09 (1.03, 1.16) 0.0053		1.08 (0.93, 1.26) 0.3076		3.31 (2.84, 3.85) <0.0001		1.09 (1.08,1.11) <0.0001	
Body mass index		0.6790		0.0464		<0.0001		0.2955
<25	1.08 (0.92, 1.27) 0.3764		1.35 (0.91, 2.00) 0.1457		2.72 (2.14, 3.45) <0.0001		1.12 (1.08,1.16) <0.0001	
25-30	1.07 (0.97, 1.18) 0.1592		0.94 (0.71, 1.23) 0.6471		2.83 (2.36, 3.40) <0.0001		1.09 (1.06,1.11) <0.0001	
≥30	1.11 (1.08, 1.15) <0.0001		1.38 (1.21, 1.57) <0.0001		1.84 (1.58, 2.14) <0.0001		1.08 (1.07,1.10) <0.0001	
Diabetes		0.4252		0.3766		0.0504		0.0486
Yes	1.08 (1.02, 1.15) 0.0107		1.04 (0.86, 1.25) 0.6992		1.89 (1.41, 2.53) 0.0001		1.05 (1.01,1.08) 0.0086	
NO	1.11 (1.04, 1.18) 0.0038		1.14 (0.97, 1.34) 0.1178		2.51 (2.22, 2.83) <0.0001		1.09 (1.07,1.10) <0.0001	
Hypertension		0.8973		0.9241		0.4959		0.6105
Yes	1.10 (1.03, 1.18) 0.0048		1.13 (0.94, 1.35) 0.1921		2.27 (1.83, 2.81) <0.0001		1.08 (1.05,1.10) <0.0001	
NO	1.10 (1.04, 1.17) 0.0034		1.12 (0.96, 1.31) 0.1624		2.47 (2.15, 2.83) <0.0001		1.08 (1.07,1.10) <0.0001	
Moderate physical activity		0.8440		0.8440		0.3402		0.8969
Yes	1.10 (1.04, 1.17) 0.0035		1.13 (0.96, 1.33) 0.1534		2.54 (2.10, 3.09) <0.0001		1.08 (1.06,1.11) <0.0001	
NO	1.10 (1.04, 1.17) 0.0034		1.11 (0.95, 1.30) 0.1798		2.31 (2.04, 2.63) <0.0001		1.08 (1.06,1.10) <0.0001	

Age, gender, race, education level, body mass index, triglyceride, glucose, poverty income ratio, hypertension, smoking, drinking, and moderate physical activity were all adjusted except the stratification variable itself. OR, odds ratio; 95% CI, 95% confidence interval; TyG-BMI, triglyceride glucose-body mass index; TyG-WC, triglyceride glucose-waist circumference; TyG-WHtR, triglyceride glucose-waist to height ratio; TyG-WWI, triglyceride glucose-weight-adjusted-waist index.

### 3.4 Smooth curve fitting between TyG-related obesity index and sarcopenia

**Figure 2:**
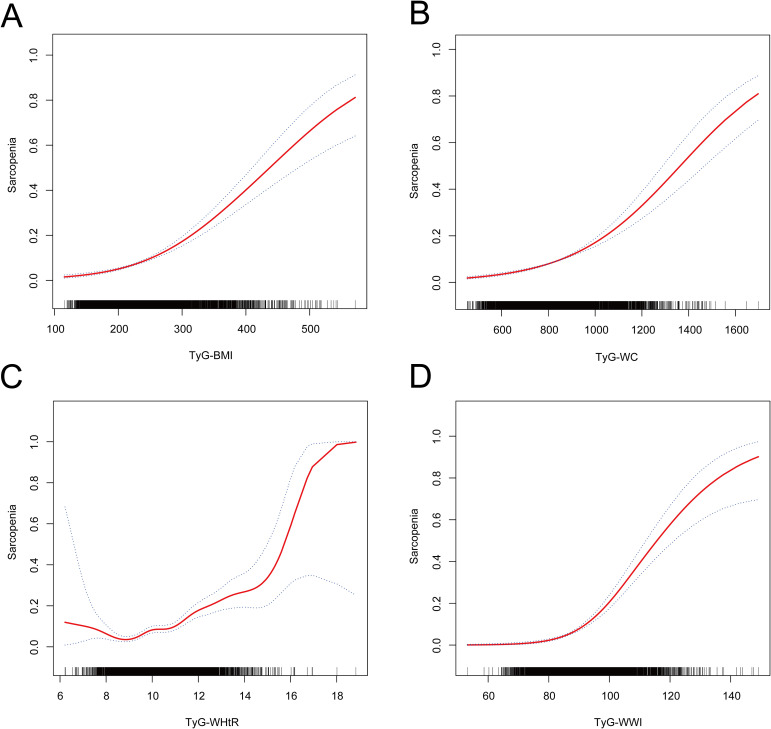
The non-linear relationship between TyG-related obesity indices and sarcopenia. The solid red line represents the smooth curve fit between variables. The blue bands represent the 95% confidence interval from the fit. Adjusted for age, gender, race, education level, body mass index, triglyceride, glucose, poverty income ratio, hypertension, smoking, drinking, and moderate physical activity. **(A)** Nonlinear relationship of TyG-BMI and sarcopenia. **(B)** Nonlinear relationship of TyG-WC and sarcopenia. **(C)** Nonlinear relationship of TyG-WHtR and sarcopenia. **(D)** Nonlinear relationship of TyG-WWI and sarcopenia.

As illustrated in **Figure 2** following the adjustment for all covariates in accordance with Model 3, a nonlinear correlation was detected among four distinct TyG-related indices of obesity and sarcopenia.

### 3.5 ROC curve of TyG index versus TyG-related obesity index levels for diagnosis of sarcopenia

**Figure 3:**
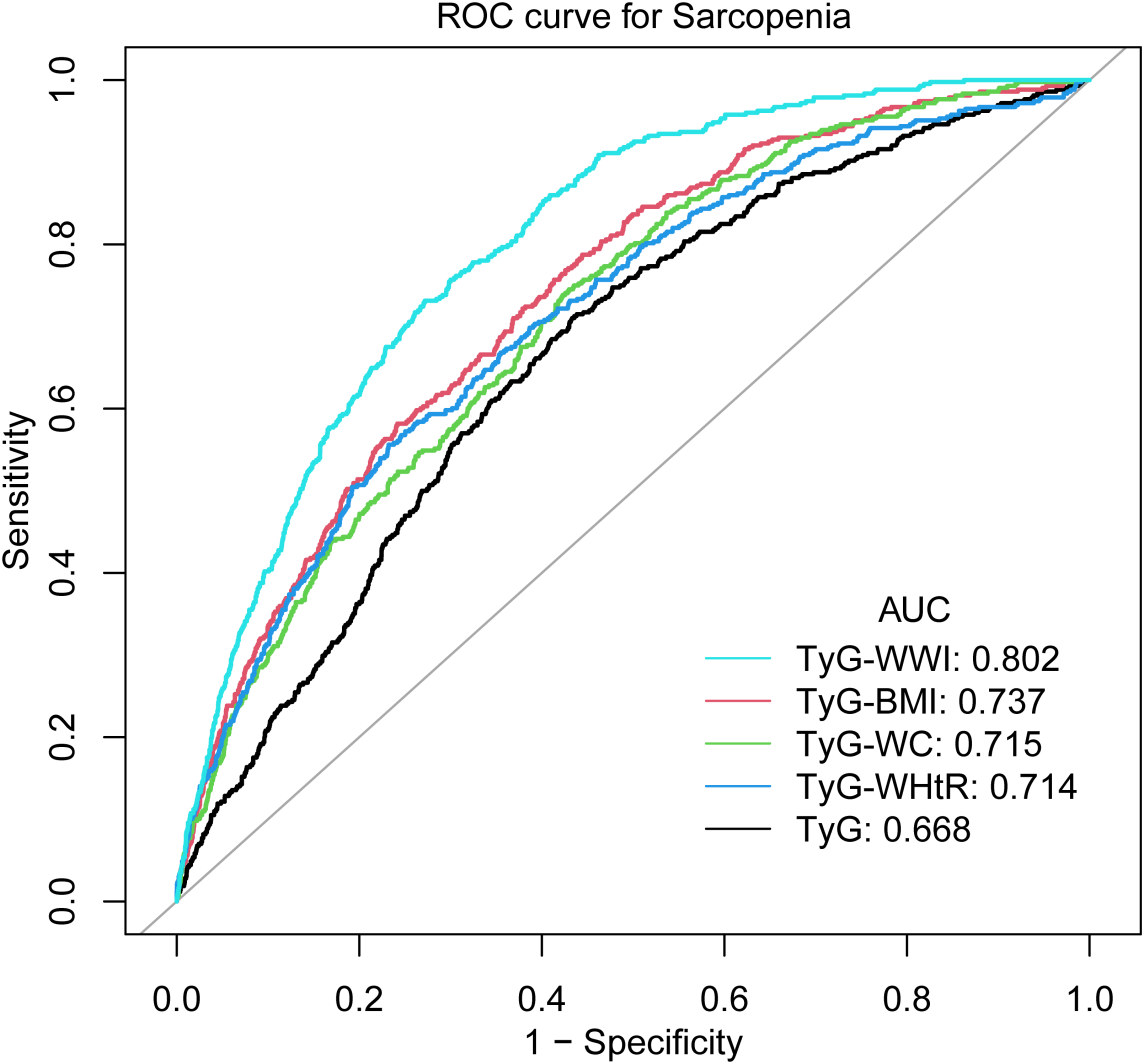
ROC curves of TyG-related obesity indices levels in diagnosing sarcopenia.

As illustrated by **Figure 3** and **Table 4**, TyG-WWI demonstrated the optimal performance, exhibiting the highest AUC value (0.8020). In comparison with other indices, the *P* values were all <0.0001, following the implementation of the FDR correction, the *P* value remained statistically significant, signifying significantly superior predictive capabilities compared to the other indices. Further analysis shows that the critical value of TyG-WWI is 97.6575, at which time the sensitivity of diagnosing sarcopenia is 0.7313 and the specificity is 0.7283. TyG-BMI exhibited the second-best predictive performance, with an AUC value of 0.7370, which surpassed the performance of TyG, TyG-WC, and TyG-WHtR. The critical value of TyG-BMI is 246.1511, at which time its sensitivity of diagnosing sarcopenia is 0.7570 and the specificity is 0.5866. The predictive performance of TyG-WC and TyG-WHtR was relatively close, with AUC values of 0.7148 and 0.7136, respectively, and there was no statistically significant difference between them (*P* value=0.9503). The predictive performance of TyG was the weakest, with the lowest AUC value (0.6680), and in compared with other indices, the *P* values were all <0.0001, following the implementation of the FDR correction, the *P* value remained statistically significant, indicating significantly worse predictive performance than other indices. Therefore, TyG-WWI is the indicator with the best predictive performance among all indicators, with the highest AUC value and statistically significant evidence superior to other models. TyG-BMI’s predictive performance was second best. TyG-WC and TyG-WHtR were also effective predictors, although not as effective as TyG-BMI and TyG-WWI. TyG exhibited the poorest predictive performance.

**Table 4:** Delong test and false discovery rate of ROC curves.

Compared index	AUC	DeLong test *P* value	False Discovery Rate *P* value
TyG	0.6680	reference	reference
TyG-WWI	0.8020	<0.0001	0.0001
TyG-BMI	0.7370	<0.0001	0.0001
TyG-WC	0.7148	<0.0001	0.0001
TyG-WHtR	0.7136	0.0019	0.0024
TyG-WWI	0.8020	reference	reference
TyG-BMI	0.7370	<0.0001	0.0001
TyG-WC	0.7148	<0.0001	0.0001
TyG-WHtR	0.7136	<0.0001	0.0001
TyG-BMI	0.7370	reference	reference
TyG-WC	0.7148	<0.0001	0.0001
TyG-WHtR	0.7136	0.2581	0.2868
TyG-WC	0.7148	reference	reference
TyG-WHtR	0.7136	0.9503	0.9503

The Benjamini-Hochberg (BH) method is employed to correct the *P* value by FDR, resulting in the *P* value of <0.0001 being assigned to 0.0001.

### 3.6 Sensitivity analysis

In **Supplementary Table 6**, this study examined the correlation between TyG-related obesity indices and ASM. In Model 3, TyG-BMI, TyG-WHtR, TyG-WWI, and TyG-WC displayed significant negative correlations with ASM. These findings indicate that increases in the four TyG-related obesity indices may correspond to decreases in muscle mass. Furthermore, the study explored the association between the TyG index and sarcopenia, as presented in **Supplementary Table 7**, revealing a significant positive correlation between the TyG index and sarcopenia.

## 4 Discussion

This cross-sectional study included 4,804 participants, with 428 diagnosed with sarcopenia. Our findings revealed a notable and positive correlation among TyG-WWI, TyG-BMI, TyG-WHtR and TyG-WC with a prevalence of sarcopenia. The aforementioned relationship remained consistent when the indices were divided into quartiles. Subgroup analysis revealed a more pronounced correlation between four indices and sarcopenia in the gender-stratified subgroup with a BMI of 30 or above. Additional ROC analysis demonstrated that the diagnostic effectiveness of four TyG-related obesity indicators was more pronounced than that of TyG. Notably, TyG-WWI demonstrated the best diagnostic efficacy in identifying individuals with sarcopenia.

Sarcopenia risk begins to escalate as early as young adulthood, with a global prevalence approximating 10% in individuals (21). Longitudinal analysis of the NHANES 1999-2006 cohort further corroborates this trend, demonstrating a significant rise in sarcopenia prevalence among adults aged 18-39 years over the study period (22). As the physical activity of young people gradually declines, there is a concomitant decrease in muscle mass and strength (23, 24). The loss of muscle mass in young people has been demonstrated to have a detrimental effect on the prevalence of sarcopenia (25). Despite the relatively low prevalence of sarcopenia in young adults, the prolonged duration of muscle loss in youth has the potential to result in elevated morbidity over time (26). Consequently, the identification and prevention of sarcopenia in young people has the potential to reduce its prevalence. Early identification and treatment of sarcopenia in young people are necessary to limit the progression of the disease and related complications (27). The present study demonstrated that TyG-WWI and TyG-BMI exhibited considerable predictive and early recognition value for sarcopenia in young and middle-aged individuals. These indicators may also contribute to delaying the progression of sarcopenia in the elderly population. Our findings demonstrate that the TyG-WWI and TyG-BMI exhibit robust predictive and discriminative capacities for identifying sarcopenia in young and middle-aged populations. These indices may further contribute to delaying the progression of geriatric sarcopenia. Notably, TyG-related obesity indicators are characterized by both low cost and ease of clinical access. This renders them particularly suitable for early screening of sarcopenia risk in middle-aged and elderly populations. The subsequent effect of this is to guide early treatment and intervention, such as resistance training (28, 29). In light of the escalating prevalence of obesity and the growing incidence of metabolic disorders among the younger population (30, 31), the prevention and monitoring of sarcopenia and its associated complications in middle-aged and young adults has become a pressing concern. At present, there has been a paucity of research conducted on this population. The findings of this study provide a basis for further research in this area, suggesting that individuals of both the young and middle-aged should observe and monitor the dynamic changes in the TyG-WWI and TyG-BMI indexes.

A number of the most recent studies corroborate our assertion. Although TyG-related obesity indices have been less well studied in relation to sarcopenia, TyG indices have been observed to be associated with low muscle mass as well as sarcopenia in various populations. Two population-based studies in Korea have established a connection between this indicator and the likelihood of low muscle mass (16, 32). The findings of Yang et al. indicated a favorable relationship between the TyG index and sarcopenia in US adults (17). Pan et al. have shown a nonlinear positive connection between TyG-BMI and sarcopenia (21). This finding is aligned with the results of our study. However, the previous research was limited in that it examined the TyG-BMI as a standalone index and did not compare it with other indications, which include the TyG indicator and those related to obesity. Our study proceeded to investigate whether the four distinct TyG-related indices of obesity exhibited a greater predictive capacity for sarcopenia compared to a single TyG index. It is noteworthy that the TyG-WWI index exhibited optimal diagnostic validity for sarcopenia, which may potentially serve as a predictor of sarcopenia.

Our subgroup analysis revealed a significant influence of gender on the relationship between TyG-WWI, TyG-WHtR, TyG-BMI, and sarcopenia. Previous studies have come to similar conclusions as ours (21). The results of some population-based surveys suggest that gender and risk with regard to sarcopenia are not entirely consistent (33, 34). Estrogen may be a factor influencing the gender differences observed in this association between males and females (35). A deficiency of estrogen has been linked to the atrophy of skeletal muscle and the accumulation of fat tissue, as well as the suppression of fatty acid beta-oxidation in both skeletal muscle and fat tissue. The development of sarcopenia is believed to be influenced by this factor (36–38). Chen et al. show that changes in estrogen levels may attenuate the correlation between the TyG index and sarcopenia in women (39). Subjects with a BMI of 30 or greater showed significant positive associations with sarcopenia concerning four indices. The relationship between sarcopenia and IR is affected by obesity, particularly in the context of sarcopenic obesity, where IR status is negatively correlated with skeletal muscle mass (40). An investigation conducted on elderly Chinese patients with diabetes revealed a heightened sarcopenia risk connected to elevated body fat levels., which supports the aforementioned findings (41). Therefore, obesity may influence the development of sarcopenia and the correlation between sarcopenia and TyG-related indices of obesity.

IR is a significant contributor to the initiation and advancement of sarcopenia. Furthermore, the extent of IR is influenced by the magnitude of sarcopenia severity, whereby the presence of obesity plays a pivotal role in this interconnection (8, 42). IR stimulates the release of sarcopenia, which in turn reduces protein synthesis in skeletal muscle and accelerates skeletal muscle protein catabolism (8). Simultaneously, sarcopenia can facilitate IR through lipotoxicity and inflammation resulting from lipid infiltration and aberrant accumulation, as well as by reducing the percentage of type I fibers in skeletal muscle (43). The results of an NHANES III-based study demonstrated that sarcopenia intensified obesity-related insulin resistance (44). This indicates the potential for a complex interrelationship between IR, obesity, and sarcopenia. At present, there is a greater understanding of the intricate relationships between IR, obesity, and sarcopenia. Prior research has indicated that individuals with sarcopenia may be predisposed to an increased risk of obesity as a consequence of their diminished activity levels (45). Obesity is often associated with a degree of inflammation. Fat accumulation often triggers an inflammatory response that leads to fat infiltration of skeletal muscle and the gradual formation of intermuscular adipose tissue (IMAT) and intracellular myocyte lipid droplets (IMCL), which are key contributors to the muscle resistance to IR (46, 47). Abnormal lipid accumulation also induces mitochondrial dysfunction, exacerbates local oxidative stress and inflammation, and contributes to the inhibition of the insulin-PI3K-mTOR pathway by protein kinase C (PKC) and p38-MAPK (48). Inflammatory cytokines have been shown to induce muscle atrophy (49), and their elevated levels impede the synthesis of insulin and insulin growth factor 1(IGF-1), further exacerbating the development of IR (50).

The present study has the following advantages: This is the inaugural study to investigate the connection among distinct TyG-related obesity indicators with sarcopenia in a US population using data from the NHANES database. Secondly, the study adjusted for potential confounding variables wherever possible and performed subgroup analysis and interacting tests to examine the impact of various elements on the relationship among the TyG-related obesity indicators with sarcopenia. Ultimately, we used ROC curves to judge the diagnostic effectiveness of individual TyG-related obesity indices and identify superior indicators compared to the TyG index, thereby establishing a novel reference for evaluating and predicting sarcopenia. It should be noted that the current study has limitations. First, the cross-sectional study design hinders the identification of causality connection among the indices with sarcopenia. Second, although this study adjusted for a considerable number of confounding variables, it was nevertheless not possible to rule out the potential impact of undetected confounders on the findings. Finally, due to an absence of data about muscle function (e.g. gait speed) in the NHANES data, the assessment of sarcopenia relied exclusively on DXA-derived body composition measures and more professional and comprehensive diagnostic criteria for sarcopenia could not be applied. Furthermore, due to the limitations of DXA, the study’s participants ranged in age from 20 to 59 years, precluding in-depth investigation of the older population. It is therefore recommended that future research employ more longitudinal studies to explore this issue in greater depth.

## 5 Conclusions

The TyG-WWI, TyG-WHtR, TyG-BMI, and TyG-WC demonstrated a nonlinear and significant positive correlation with a prevalence of sarcopenia. Furthermore, these four indices exhibited superior diagnostic accuracy in detecting sarcopenia compared to the TyG index. Notably, we found optimal diagnostic validity of TyG-WWI for sarcopenia. This indicates that the TyG-related obesity indices, specifically the TyG-WWI index, have potential as reliable indicators for assessing and predicting sarcopenia.

## Data Availability

The original contributions presented in the study are included in the article/**Supplementary Material**. Further inquiries can be directed to the corresponding author/s.
