# Supplementary material for: Associations between different triglyceride glucose index-related obesity indices and sarcopenia: a cross-sectional study
Source: Front Endocrinol (Lausanne). 2025 May 29;16:1511232. doi: 10.3389/fendo.2025.1511232 (PMC12158711; doi:10.3389/fendo.2025.1511232)
Supplement: Supplementary file 1 [file Table1.docx]

| Covariates | Definition |
| --- | --- |
| Smoking status |  |
| Yes | smoke more than 100 cigarettes in life and smoke some days or every day |
| No | smoked less than 100 cigarettes in life |
| Drinking status |  |
| Yes | drink a minimum of 12 glasses of various kinds of alcohol each year |
| No | drink less than 12 glasses of various kinds of alcohol each year |
| Hypertension status | self-reported doctor's diagnosis of hypertension |
| Diabetes status | self-reported a doctor-diagnosed diabetes |
| Moderate physical activity |  |
| Yes | do any moderate-intensity sports, fitness, or recreational activities that cause a small increase in breathing or heart rate such as brisk walking, bicycling, swimming, or volleyball for at least 10 minutes continuously |
| No | do any moderate-intensity sports, fitness, or recreational activities that cause a small increase in breathing or heart rate such as brisk walking, bicycling, swimming, or volleyball for less than 10 minutes continuously |

**Supplementary Table 1 Definition of covariates**

**Supplementary Table 2 Baseline characteristics according to TyG-BMI quartiles**

|  | Quartile1  Mean(95%CI) | Quartile2  Mean(95%CI) | Quartile3  Mean(95%CI) | Quartile4  Mean(95%CI) | *P*-value |
| --- | --- | --- | --- | --- | --- |
| Age(years) | 35.68 (34.62,36.74) | 40.00 (39.04,40.97) | 41.30 (40.57,42.03) | 41.29 (40.37,42.21) | <0.0001 |
| Body mass index (%) | 21.64 (21.47,21.81) | 25.97 (25.80,26.14) | 29.77 (29.58,29.97) | 37.40 (36.97,37.84) | <0.0001 |
| Poverty income ratio (%) | 2.87  (2.69,3.05) | 2.98 (2.82,3.13) | 2.85 (2.70,3.01) | 2.63 (2.50,2.76) | 0.0010 |
| Triglyceride (mg/dl) | 66.47 (63.88,69.06) | 97.35 (93.37,101.34) | 136.33 (130.89,141.78) | 182.31 (168.18,196.45) | <0.0001 |
| Glucose (mg/dl) | 94.09 (93.18,94.99) | 99.11 (98.18,100.04) | 105.37 (103.18,107.57) | 119.64 (116.73,122.55) | <0.0001 |
| Gender |  |  |  |  | <0.0001 |
| Male | 39.25 (35.89,42.71) | 55.96 (51.86,59.98) | 59.80 (56.10,63.38) | 47.34 (43.95,50.76) |  |
| Female | 60.75 (57.29,64.11) | 44.04 (40.02,48.14) | 40.20 (36.62,43.90) | 52.66 (49.24,56.05) |  |
| Race(%) |  |  |  |  | <0.0001 |
| Mexican American | 5.26  (4.04,6.83) | 9.63 (7.43,12.39) | 13.74 (10.90,17.18) | 13.78 (10.61,17.71) |  |
| Other Hispanic | 6.80  (4.91,9.33) | 6.91 (5.04,9.40) | 8.78 (6.63,11.54) | 7.56 (6.03,9.43) |  |
| Non-Hispanic White | 63.43 (58.50,68.10) | 62.13(57.35,66.69) | 60.47 (55.55,65.19) | 60.91 (55.58,65.98) |  |
| Non-Hispanic Black | 10.12 (7.93,12.84) | 10.45 (8.45,12.86) | 9.13 (7.35,11.29) | 12.76 (10.30,15.71) |  |
| Other Race | 14.39 (12.16,16.95) | 10.89 (8.70,13.55) | 7.88 (6.30,9.82) | 4.99 (3.60,6.88) |  |
| Education level (%) |  |  |  |  | 0.0338 |
| Less than high school | 11.43 (8.86,14.63) | 12.66 (10.12,15.72) | 15.53 (13.00,18.46) | 16.30 (13.69,19.29) |  |
| High school diploma | 18.80 (15.50,22.62) | 22.66 (18.60,27.31) | 21.40 (17.91,25.35) | 23.44 (20.37,26.83) |  |
| More than high school | 69.77 (64.57,74.50) | 64.68 (59.26,69.75) | 63.01 (58.23,67.54) | 60.26 (56.62,63.79) |  |
| Others | 0.00  (0.00,0.00) | 0.00 (0.00,0.00) | 0.07(0.01,0.49) | 0.00 (0.00,0.00) |  |
| Hypertension status (%) |  |  |  |  | <0.0001 |
| Yes | 9.86 (7.50,12.86) | 18.84 (16.15,21.86) | 27.16 (23.78,30.82) | 38.10 (34.32,42.03) |  |
| No | 90.14 (87.14,92.50) | 81.16 (78.14,83.85) | 72.84 (69.18,76.22) | 61.90 (57.97,65.68) |  |
| Smoking status (%) |  |  |  |  | 0.1988 |
| Yes | 38.76 (34.02,43.74) | 43.07 (38.97,47.27) | 42.61 (39.23,46.06) | 44.40 (40.22,48.66) |  |
| No | 61.24 (56.26,65.98) | 56.93 (52.73,61.03) | 57.39 (53.94,60.77) | 55.60 (51.34,59.78) |  |
| Drinking status (%) |  |  |  |  | 0.0460 |
| Yes | 79.60 (76.49,82.39) | 82.36 (79.20,85.13) | 80.07 (77.22,82.64) | 77.17 (73.71,80.29) |  |
| No | 20.40 (17.61,23.51) | 17.64 (14.87,20.80) | 19.93 (17.36,22.78) | 22.83 (19.71,26.29) |  |
| Diabetes status (%) |  |  |  |  | <0.0001 |
| Yes | 1.04  (0.57,1.89) | 2.39 (1.55,3.66) | 4.85 (3.66,6.40) | 15.36 (13.15,17.85) |  |
| No | 98.96 (98.11,99.43) | 97.61 (96.34,98.45) | 95.15 (93.60,96.34) | 84.64 (82.15,86.85) |  |
| Moderate physical activity (%) |  |  |  |  | 0.0001 |
| Yes | 54.21 (49.70,58.65) | 49.71 (45.57,53.86) | 44.73 (40.92,48.60) | 42.32 (38.68,46.05) |  |
| No | 45.79 (41.35,50.30) | 50.29 (46.14,54.43) | 55.27 (51.40,59.08) | 57.68 (53.95,61.32) |  |
| Sarcopenia |  |  |  |  | <0.0001 |
| No | 98.70 (98.12,99.10) | 96.44 (95.00,97.47) | 92.93 (89.97,95.06) | 82.08 (78.62,85.09) |  |
| Yes | 1.30  (0.90,1.88) | 3.56 (2.53,5.00) | 7.07 (4.94,10.03) | 17.92 (14.91,21.38) |  |

For continuous variables: survey-weighted mean (95% CI), *P*-value was by survey-weighted linear regression. For categorical variables: survey-weighted percentage (95% CI), *P*-value was by survey-weighted Chi-square test. TyG-BMI, triglyceride glucose-body mass index.

**Supplementary Table 3 Baseline characteristics according to TyG-WC quartiles**

|  | Quartile1  Mean(95%CI) | Quartile2  Mean(95%CI) | Quartile3  Mean(95%CI) | Quartile4  Mean(95%CI) | *P*-value |
| --- | --- | --- | --- | --- | --- |
| Age(years) | 34.13(33.03,35.23) | 39.35(38.33,40.37) | 41.89 (41.21,42.57) | 42.65(41.78,43.52) | <0.0001 |
| Body mass index (%) | 22.12(21.91,22.34) | 26.23(25.97,26.50) | 29.82 (29.54,30.11) | 36.09 (35.57,36.62) | <0.0001 |
| Poverty income ratio (%) | 2.84 (2.69,2.98) | 2.97 (2.80,3.15) | 2.89 (2.72,3.06) | 2.63 (2.50,2.77) | 0.0023 |
| Triglyceride (mg/dl) | 62.07(59.84,64.30) | 93.00(89.82,96.17) | 125.27(120.44,130.09) | 197.86(183.68,212.04) | <0.0001 |
| Glucose (mg/dl) | 93.14 (92.46,93.82) | 98.69(97.68,99.69) | 103.16(101.90,104.41) | 122.32 (119.09,125.55) | <0.0001 |
| Gender |  |  |  |  | <0.0001 |
| Male | 36.15 (32.46,40.01) | 49.62(45.29,53.95) | 58.31 (54.61,61.92) | 57.85 (54.69,60.95) |  |
| Famale | 63.85 (59.99,67.54) | 50.38(46.05,54.71) | 41.69 (38.08,45.39) | 42.15 (39.05,45.31) |  |
| Race(%) |  |  |  |  | <0.0001 |
| Mexican American | 6.08 (4.53,8.12) | 10.26 (7.86,13.27) | 12.86 (10.31,15.92) | 12.93 (9.93,16.68) |  |
| Other Hispanic | 7.34 (5.19,10.29) | 7.87 (5.72,10.72) | 7.69 (6.03,9.77) | 7.11 (5.48,9.18) |  |
| Non-Hispanic White | 59.67 (54.23,64.89) | 60.33(55.63,64.84) | 62.13 (57.50,66.55) | 64.73 (59.84,69.34) |  |
| Non-Hispanic Black | 12.66 (10.02,15.87) | 10.24 (8.47,12.34) | 9.03 (7.15,11.34) | 10.58 (8.42,13.21) |  |
| Other Race | 14.24 (11.95,16.88) | 11.30 (8.97,14.15) | 8.29 (6.79,10.08) | 4.64 (3.60,5.96) |  |
| Education level (%) |  |  |  |  | 0.0041 |
| Less than high school | 10.65 (8.34,13.52) | 14.04 (11.33,17.28) | 14.47 (11.81,17.62) | 16.53 (13.86,19.59) |  |
| High school diploma | 18.05 (14.67,22.00) | 21.67(18.21,25.57) | 22.46 (19.11,26.21) | 23.98 (20.57,27.75) |  |
| More than high school | 71.30 (66.57,75.61) | 64.22(59.09,69.05) | 63.07 (58.52,67.39) | 59.50 (54.99,63.85) |  |
| Others | 0.00 (0.00,0.00) | 0.07 (0.01,0.49) | 0.00 (0.00,0.00) | 0.00 (0.00,0.00) |  |
| Hypertension status (%) |  |  |  |  | <0.0001 |
| Yes | 7.87 (5.72,10.72) | 18.82 (16.07,21.92) | 27.20 (23.99,30.66) | 39.01 (35.01,43.16) |  |
| No | 92.13 (89.28,94.28) | 81.18 (78.08,83.93) | 72.80 (69.34,76.01) | 60.99 (56.84,64.99) |  |
| Smoking status (%) |  |  |  |  | <0.0001 |
| Yes | 34.37 (29.94,39.08) | 41.85 (37.61,46.22) | 44.27 (40.93,47.67) | 48.00 (43.50,52.53) |  |
| No | 65.63 (60.92,70.06) | 58.15 (53.78,62.39) | 55.73 (52.33,59.07) | 52.00 (47.47,56.50) |  |
| Drinking status (%) |  |  |  |  | 0.8820 |
| Yes | 79.24 (75.92,82.21) | 80.61 (78.04,82.95) | 80.00 (77.24,82.50) | 79.44 (75.56,82.85) |  |
| No | 20.76 (17.79,24.08) | 19.39 (17.05,21.96) | 20.00 (17.50,22.76) | 20.56 (17.15,24.44) |  |
| Diabetes status (%) |  |  |  |  | <0.0001 |
| Yes | 0.56 (0.27,1.13) | 2.04 (1.32,3.14) | 4.48 (3.39,5.90) | 16.06 (13.83,18.58) |  |
| No | 99.44 (98.87,99.73) | 97.96 (96.86,98.68) | 95.52 (94.10,96.61) | 83.94 (81.42,86.17) |  |
| Moderate physical activity (%) |  |  |  |  | <0.0001 |
| Yes | 55.96 (51.46,60.37) | 49.76 (45.13,54.39) | 43.92 (39.93,47.99) | 41.83 (38.56,45.18) |  |
| No | 44.04 (39.63,48.54) | 50.24 (45.61,54.87) | 56.08 (52.01,60.07) | 58.17 (54.82,61.44) |  |
| Sarcopenia |  |  |  |  | <0.0001 |
| No | 98.86 (98.14,99.30) | 95.48 (93.94,96.63) | 92.37 (89.27,94.62) | 84.02 (80.96,86.67) |  |
| Yes | 1.14 (0.70,1.86) | 4.52 (3.37,6.06) | 7.63 (5.38,10.73) | 15.98 (13.33,19.04) |  |

For continuous variables: survey-weighted mean (95% CI), *P*-value was by survey-weighted linear regression. For categorical variables: survey-weighted percentage (95% CI), *P*-value was by survey-weighted Chi-square test. TyG-WC, triglyceride glucose-waist circumference.

**Supplementary Table 4 Baseline characteristics according to TyG-WHtR quartiles**

|  | Quartile1  Mean(95%CI) | Quartile2  Mean(95%CI) | Quartile3  Mean(95%CI) | Quartile4  Mean(95%CI) | *P*-value |
| --- | --- | --- | --- | --- | --- |
| Age(years) | 35.85(34.98,36.71) | 38.71 (37.86,39.55) | 41.05 (40.10,42.01) | 43.48 (42.70,44.27) | <0.0001 |
| Body mass index (%) | 31.27 (30.68,31.85) | 28.90 (28.36,29.44) | 27.51 (27.08,27.95) | 26.41 (25.91,26.91) | <0.0001 |
| Poverty income ratio (%) | 3.02 (2.86,3.18) | 2.85 (2.68,3.01) | 2.83 (2.66,3.00) | 2.60 (2.46,2.74) | <0.0001 |
| Triglyceride (mg/dl) | 76.69 (73.05,80.33) | 103.20(98.43,107.97) | 133.42(126.55,140.29) | 179.35 (163.93,194.78) | <0.0001 |
| Glucose (mg/dl) | 99.06(97.94,100.17) | 100.85(99.50,102.20) | 104.59(102.81,106.38) | 115.36(111.28,119.43) | <0.0001 |
| Gender |  |  |  |  | <0.0001 |
| Male | 66.77 (63.76,69.65) | 56.32 (52.46,60.11) | 48.38 (44.17,52.61) | 26.47 (23.26,29.95) |  |
| Famale | 33.23 (30.35,36.24) | 43.68 (39.89,47.54) | 51.62 (47.39,55.83) | 73.53 (70.05,76.74) |  |
| Race(%) |  |  |  |  | <0.0001 |
| Mexican American | 6.87 (4.92,9.51) | 8.90 (6.64,11.82) | 12.62 (10.18,15.55) | 14.87 (11.77,18.60) |  |
| Other Hispanic | 5.15 (3.59,7.34) | 6.88 (5.12,9.18) | 8.30 (6.32,10.82) | 10.26 (7.86,13.28) |  |
| Non-Hispanic White | 64.70 (59.25,69.79) | 64.11 (58.91,68.99) | 61.39 (56.57,65.99) | 55.60 (49.92,61.14) |  |
| Non-Hispanic Black | 18.57 (15.27,22.40) | 11.49 (9.50,13.83) | 7.05 (5.61,8.82) | 3.85 (2.79,5.27) |  |
| Other Race | 4.71 (3.72,5.95) | 8.63 (6.51,11.37) | 10.64 (8.65,13.03) | 15.43 (12.59,18.77) |  |
| Education level (%) |  |  |  |  | <0.0001 |
| Less than high school | 9.34 (7.44,11.66) | 10.76 (8.43,13.63) | 15.63 (13.01,18.66) | 21.65 (17.69,26.21) |  |
| High school diploma | 21.27 (17.95,25.03) | 19.97 (16.71,23.70) | 21.66 (18.56,25.11) | 23.82 (19.35,28.95) |  |
| More than high school | 69.39 (64.91,73.52) | 69.21 (64.40,73.63) | 62.72 (57.86,67.33) | 54.53 (49.40,59.57) |  |
| Others | 0.00 (0.00,0.00) | 0.06 (0.01,0.46) | 0.00 (0.00,0.00) | 0.00 (0.00,0.00) |  |
| Hypertension status (%) |  |  |  |  | 0.5112 |
| Yes | 22.45 (18.95,26.40) | 22.25 (18.85,26.06) | 23.68 (20.53,27.14) | 25.66 (22.23,29.43) |  |
| No | 77.55 (73.60,81.05) | 77.75 (73.94,81.15) | 76.32 (72.86,79.47) | 74.34 (70.57,77.77) |  |
| Smoking status (%) |  |  |  |  | 0.0063 |
| Yes | 37.99 (33.73,42.44) | 40.50 (36.57,44.56) | 44.87 (41.09,48.72) | 46.42 (42.07,50.82) |  |
| No | 62.01 (57.56,66.27) | 59.50 (55.44,63.43) | 55.13 (51.28,58.91) | 53.58 (49.18,57.93) |  |
| Drinking status (%) |  |  |  |  | <0.0001 |
| Yes | 83.10 (79.90,85.88) | 81.48 (78.45,84.17) | 81.48 (77.99,84.53) | 71.85 (67.78,75.59) |  |
| No | 16.90 (14.12,20.10) | 18.52 (15.83,21.55) | 18.52 (15.47,22.01) | 28.15 (24.41,32.22) |  |
| Diabetes status (%) |  |  |  |  | <0.0001 |
| Yes | 3.67 (2.45,5.47) | 3.25 (2.17,4.82) | 5.14 (3.86,6.82) | 12.52 (9.80,15.87) |  |
| No | 96.33 (94.53,97.55) | 96.75 (95.18,97.83) | 94.86 (93.18,96.14) | 87.48 (84.13,90.20) |  |
| Moderate physical activity (%) |  |  |  |  | 0.0209 |
| Yes | 52.22 (48.06,56.36) | 48.42 (43.95,52.91) | 43.98 (40.09,47.94) | 45.92 (42.09,49.79) |  |
| No | 47.78 (43.64,51.94) | 51.58 (47.09,56.05) | 56.02 (52.06,59.91) | 54.08 (50.21,57.91) |  |
| Sarcopenia |  |  |  |  | <0.0001 |
| No | 98.00 (96.90,98.72) | 93.55 (91.26,95.27) | 92.55 (90.64,94.10) | 84.87 (81.38,87.80) |  |
| Yes | 2.00 (1.28,3.10) | 6.45 (4.73,8.74) | 7.45 (5.90,9.36) | 15.13 (12.20,18.62) |  |

For continuous variables: survey-weighted mean (95% CI), *P*-value was by survey-weighted linear regression. For categorical variables: survey-weighted percentage (95% CI), *P*-value was by survey-weighted Chi-square test. TyG-WHtR, triglyceride glucose-waist-to-height ratio.

**Supplementary Table 5 Baseline characteristics according to TyG-WWI quartiles**

|  | Quartile1  Mean(95%CI) | Quartile2  Mean(95%CI) | Quartile3  Mean(95%CI) | Quartile4  Mean(95%CI) | *P*-value |
| --- | --- | --- | --- | --- | --- |
| Age(years) | 33.55(32.50,34.61) | 38.62(37.65,39.60) | 41.63 (40.90,42.36) | 44.77 (43.98,45.55) | <0.0001 |
| Body mass index (%) | 24.13(23.73,24.53) | 27.11(26.77,27.45) | 30.31 (29.87,30.75) | 33.30 (32.72,33.88) | <0.0001 |
| Poverty income ratio (%) | 2.93 (2.80,3.05) | 2.93 (2.75,3.10) | 2.90 (2.75,3.04) | 2.58 (2.44,2.71) | <0.0001 |
| Triglyceride (mg/dl) | 56.82(55.17,58.46) | 86.69(84.33,89.06) | 125.72(120.81,130.63) | 217.24 (203.57,230.91) | <0.0001 |
| Glucose (mg/dl) | 93.50(92.73,94.26) | 98.24(97.32,99.16) | 102.12(101.01,103.23) | 125.12 (121.61,128.63) | <0.0001 |
| Gender |  |  |  |  | 0.8578 |
| Male | 49.85 (46.18,53.52) | 50.09 (46.67,53.50) | 51.79 (48.14,55.43) | 50.89 (47.29,54.48) |  |
| Famale | 50.15 (46.48,53.82) | 49.91 (46.50,53.33) | 48.21 (44.57,51.86) | 49.11 (45.52,52.71) |  |
| Race(%) |  |  |  |  | <0.0001 |
| Mexican American | 5.29 (3.86,7.20) | 10.08 (7.63,13.20) | 12.18 (9.79,15.05) | 15.06 (11.77,19.06) |  |
| Other Hispanic | 5.86 (4.01,8.50) | 8.27 (6.34,10.73) | 7.54 (5.94,9.54) | 8.39 (6.38,10.96) |  |
| Non-Hispanic White | 61.90 (56.40,67.12) | 61.72 (56.59,66.60) | 60.33 (56.03,64.48) | 63.08 (57.95,67.93) |  |
| Non-Hispanic Black | 16.27 (13.21,19.88) | 10.38 (8.35,12.82) | 9.38 (7.76,11.29) | 6.14 (4.64,8.08) |  |
| Other Race | 10.67 (8.95,12.69) | 9.55 (7.42,12.22) | 10.57 (8.59,12.94) | 7.34 (6.08,8.83) |  |
| Education level (%) |  |  |  |  | <0.0001 |
| Less than high school | 9.43 (7.25,12.19) | 10.91 (8.59,13.76) | 14.88 (11.98,18.33) | 21.04 (18.25,24.12) |  |
| High school diploma | 19.22 (15.61,23.45) | 20.06 (16.85,23.72) | 22.77 (20.06,25.72) | 24.43 (19.90,29.62) |  |
| More than high school | 71.34 (66.14,76.03) | 68.96 (64.78,72.86) | 62.36 (58.43,66.13) | 54.53 (49.71,59.27) |  |
| Others | 0.00 (0.00,0.00) | 0.07 (0.01,0.49) | 0.00 (0.00,0.00) | 0.00 (0.00,0.00) |  |
| Hypertension status (%) |  |  |  |  | <0.0001 |
| Yes | 10.52 (7.86,13.95) | 17.08 (14.79,19.63) | 28.22 (24.53,32.23) | 38.71 (34.79,42.77) |  |
| No | 89.48 (86.05,92.14) | 82.92 (80.37,85.21) | 71.78 (67.77,75.47) | 61.29 (57.23,65.21) |  |
| Smoking status (%) |  |  |  |  | <0.0001 |
| Yes | 36.00 (31.11,41.20) | 36.41 (32.47,40.53) | 44.22 (40.47,48.03) | 52.86 (48.49,57.19) |  |
| No | 64.00 (58.80,68.89) | 63.59 (59.47,67.53) | 55.78 (51.97,59.53) | 47.14 (42.81,51.51) |  |
| Drinking status (%) |  |  |  |  | 0.0267 |
| Yes | 81.75 (78.99,84.22) | 81.14 (78.26,83.71) | 79.43 (76.45,82.11) | 76.83 (73.19,80.11) |  |
| No | 18.25 (15.78,21.01) | 18.86 (16.29,21.74) | 20.57 (17.89,23.55) | 23.17 (19.89,26.81) |  |
| Diabetes status (%) |  |  |  |  | <0.0001 |
| Yes | 0.96 (0.49,1.88) | 1.48 (0.95,2.29) | 3.83 (2.73,5.34) | 17.78 (15.55,20.26) |  |
| No | 99.04 (98.12,99.51) | 98.52 (97.71,99.05) | 96.17 (94.66,97.27) | 82.22 (79.74,84.45) |  |
| Moderate physical activity (%) |  |  |  |  | <0.0001 |
| Yes | 56.98 (52.60,61.25) | 48.41 (44.17,52.68) | 46.85 (43.21,50.52) | 38.33 (34.42,42.38) |  |
| No | 43.02 (38.75,47.40) | 51.59 (47.32,55.83) | 53.15 (49.48,56.79) | 61.67 (57.62,65.58) |  |
| Sarcopenia |  |  |  |  | <0.0001 |
| No | 99.63 (99.31,99.80) | 97.83 (96.79,98.54) | 92.77 (90.42,94.57) | 79.45 (75.71,82.75) |  |
| Yes | 0.37 (0.20,0.69) | 2.17 (1.46,3.21) | 7.23 (5.43,9.58) | 20.55 (17.25,24.29) |  |

For continuous variables: survey-weighted mean (95% CI), *P*-value was by survey-weighted linear regression. For categorical variables: survey-weighted percentage (95% CI), *P*-value was by survey-weighted Chi-square test. TyG-WWI, triglyceride glucose-weight-adjusted waist index.

## Supplementary Table 6 The correlation between TyG-related obesity indices and ASM

| ASM | n  Mean  (95%CI) | Model 1 | Model 2 | Model 3 |
| --- | --- | --- | --- | --- |
|  |  | β  (95%CI)  *P*-value | β  (95%CI)  *P*-value | β  (95%CI)  *P*-value |
| TyG-BMI | 4804  22.904  (22.650 ,23.157) | 0.482  (0.456, 0.508)  <0.0001 | 0.485  (0.466, 0.505)  <0.0001 | -0.009  (-0.009, -0.008)  <0.0001 |
| TyG-WC | 4804  22.904  (22.650 ,23.157) | 1.890  (1.783,1.997)  <0.0001 | 1.702  (1.618, 1.787)  <0.0001 | -0.033  (-0.036, -0.030)  <0.0001 |
| TyG-WHtR | 4804  22.904  (22.650 ,23.157) | -2.744  (-2.864, -2.625)  <0.0001 | -2.028  (-2.144, -1.912)  <0.0001 | -0.022  (-0.025, -0.019)  <0.0001 |
| TyG-WWI | 4804  22.904  (22.650 ,23.157) | 0.069  (0.051, 0.086)  <0.0001 | 0.105  (0.091, 0.119)  <0.0001 | -0.008  (-0.009, -0.007)  <0.0001 |

95%CI: survey-weighted percentage. Model 1 did not incorporate the use of covariates. Model 2 incorporates adjustments for gender, age, and race. Model 3 incorporates adjustments for race, education level, triglyceride, glucose, poverty income ratio, hypertension, smoking, drinking, and moderate physical activity. TyG–BMI was treated as a continuous variable with per 10-unit increase. TyG–WC was treated as a continuous variable with a per 100-unit increase. Abbreviations: TyG-BMI, triglyceride glucose-body mass index; TyG-WC, triglyceride glucose-waist circumference; TyG-WHtR, triglyceride glucose-waist to height ratio; TyG-WWI, triglyceride glucose-weight-adjusted-waist index, Ref., reference.

**Supplementary Table 7 The association between the TyG index and sarcopenia**

| Sarcopenia | n  Mean(95%CI) | Model 1 | Model 2 | Model 3 |
| --- | --- | --- | --- | --- |
|  |  | OR (95%CI)  *P*-value | OR (95%CI)  *P*-value | OR (95%CI)  *P*-value |
| TyG | 4804  7.391 (6.133 ,8.649) | 2.115 (1.848, 2.422) <0.0001 | 1.792 (1.545, 2.079) <0.0001 | 2.703 (1.676, 4.359) 0.0002 |
| TyG quartile |  |  |  |  |
| Q1 | 1200  2.421(1.449,3.394) | Ref. | Ref. | Ref. |
| Q2 | 1201  4.783 (3.123,6.442) | 2.024 (1.242, 3.298) 0.0063 | 1.734 (1.067, 2.819) 0.0305 | 1.665 (1.038, 2.669) 0.0403 |
| Q3 | 1202  8.632 (6.435,10.828) | 3.807 (2.373, 6.109) <0.0001 | 2.911 (1.826, 4.639) <0.0001 | 2.662 (1.674, 4.235) 0.0002 |
| Q4 | 1201  13.681(10.891,16.471) | 6.387 (4.019, 10.150) <0.0001 | 4.468 (2.798, 7.135) <0.0001 | 3.811 (2.227, 6.522) <0.0001 |
| *P* for trend |  | <0.0001 | <0.0001 | <0.0001 |

95%CI: survey-weighted percentage. For sarcopenia: survey-weighted OR (95%CI) *P*-value. Model 1 did not incorporate the use of covariates. Model 2 incorporates adjustments for gender, age, and race. Model 3 incorporates adjustments for race, education level, triglyceride, glucose, poverty income ratio, hypertension, smoking, drinking, and moderate physical activity. Abbreviations: TyG, triglyceride glucose index.
